# Genomic and transcriptomic alterations following intergeneric hybridization and polyploidization in the *Chrysanthemum nankingense*×*Tanacetum vulgare* hybrid and allopolyploid (Asteraceae)

**DOI:** 10.1038/s41438-017-0003-0

**Published:** 2018-02-07

**Authors:** Xiangyu Qi, Haibin Wang, Aiping Song, Jiafu Jiang, Sumei Chen, Fadi Chen

**Affiliations:** Key Laboratory of Landscape Agriculture, College of Horticulture, Nanjing Agricultural University, Ministry of Agriculture, Nanjing, 210095 China

## Abstract

Allopolyploid formation involves two major events: interspecific hybridization and polyploidization. A number of species in the Asteraceae family are polyploids because of frequent hybridization. The effects of hybridization on genomics and transcriptomics in *Chrysanthemum nankingense×Tanacetum vulgare* hybrids have been reported. In this study, we obtained allopolyploids by applying a colchicine treatment to a synthesized *C. nankingense*×*T. vulgare* hybrid. Sequence-related amplified polymorphism (SRAP), methylation-sensitive amplification polymorphism (MSAP), and high-throughput RNA sequencing (RNA-Seq) technologies were used to investigate the genomic, epigenetic, and transcriptomic alterations in both the hybrid and allopolyploids. The genomic alterations in the hybrid and allopolyploids mainly involved the loss of parental fragments and the gain of novel fragments. The DNA methylation level of the hybrid was reduced by hybridization but was restored somewhat after polyploidization. There were more significant differences in gene expression between the hybrid/allopolyploid and the paternal parent than between the hybrid/allopolyploid and the maternal parent. Most differentially expressed genes (DEGs) showed down-regulation in the hybrid/allopolyploid relative to the parents. Among the non-additive genes, transgressive patterns appeared to be dominant, especially repression patterns. Maternal expression dominance was observed specifically for down-regulated genes. Many methylase and methyltransferase genes showed differential expression between the hybrid and parents and between the allopolyploid and parents. Our data indicate that hybridization may be a major factor affecting genomic and transcriptomic changes in newly formed allopolyploids. The formation of allopolyploids may not simply be the sum of hybridization and polyploidization changes but also may be influenced by the interaction between these processes.

## Genome evolution: Complex interactions in the composites

Two drivers of plant speciation – hybridization and polyploidization – may interact to cause significant genetic and genomic changes. The creation of new species and varieties of plant, both in nature and in artificial breeding, commonly employs the processes of hybridization (breeding two different species together) and polyploidization (multiplication of the entire genome). When the two processes occur together, an ‘allopolyploid’ is created. A team at Nanjing Agricultural University, led by Fadi Chen, explored the genomic changes associated with allopolyploidization in a hybrid of two species of composites (Asteraceae, the daisy family), chrysanthemum and tansy. Using a combination of modern genetic approaches, they found that differences in genetic makeup, gene activity, and molecular modification of the genome were associated with both hybridization and polyploidization and, potentially, with an interaction between the two.

## Introduction

Hybridization has contributed to the evolution of higher plants, and it is considered to be a potent evolutionary force driving genetic variation and functional novelty.^1–3^ Hybridization occurs frequently in flowering plants and is considered a useful tool to aid in importing desirable genes and traits into hybrids, resulting in hybrids with superior phenotypes and hybrids that may have undergone directional and rapid changes in their evolutionary history^4,5^. Polyploidization has also had an important role in plant evolution and speciation^1^. It is believed that all angiosperms underwent at least one round of polyploidization during their evolution^6–8^. On the basis of the mode of origin, polyploids are divided into two forms: an autopolyploid is derived from an intraspecies genome duplication event, whereas an allopolyploid originates from hybridization between different species followed by genome doubling or the fusion of unreduced gametes between species; allopolyploidy is the most common type of polyploidy^9^.

Molecular markers, microarray data, and high-throughput RNA sequencing (RNA-Seq) have been used to study genomic and transcriptomic changes in allopolyploids^10–14^. To determine the adjustment of duplicated genes and genomes during the early stages of polyploidization, many studies have used artificially synthesized polyploid materials using molecular markers and RNA-Seq technologies^11,13–15^. Hybridization appears to be often accompanied by changes to genomic sequences, the epigenome, and the patterns of gene transcript levels^16–18^. Different allopolyploids exhibit dynamic and pervasive changes in the genome sequence, including DNA sequence elimination^19,20^, transposon activation^21,22^, genome rearrangement^23^, and gene silencing^24^. Recent studies have indicated that allopolyploid formation is accompanied by extensive alterations in parental gene expression (“transcriptome shock”)^12–14,25^, which is likely the result of interspecific hybridization rather than polyploidization^26^.

The Asteraceae genus *Chrysanthemum* includes ploidy states ranging from diploid to decaploid^27^. Although numerous studies have provided valuable information about the rapid genomic and transcriptomic changes in many other plants, little is known about these changes in Asteraceae^28^. In an earlier study, an intergeneric hybrid was successfully created between *C. nankingense* and *T. vulgare*^29^. DNA-amplified fragment length polymorphism (AFLP) and methylation-sensitive amplification polymorphism (MSAP) techniques were used to detect genomic and epigenomic changes, and cDNA-AFLP was applied to characterize transcriptomic changes in the newly synthesized *C. nankingense*×*T. vulgare* hybrids^30^. In the present study, we obtained allopolyploids of these hybrids after colchicine treatment of a synthesized *C. nankingense*×*T. vulgare* hybrid. To further clarify the genome evolution of these plants, we detected genomic and epigenetic changes induced by intergeneric hybridization and polyploidization using sequence-related amplified polymorphism (SRAP) and MSAP marker technologies and investigated the relative transcript impacts of hybridization and polyploidization by applying RNA-Seq to compare the transcriptomes of the hybrid, allopolyploid, and parents.

## Materials and methods

### Plant materials

**Fig. 1:**
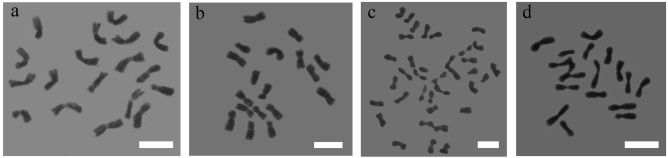
Mitotic chromosomes of materials. **a** *C. nankingense*. **b** Hybrid. **c** Allopolyploid. **d** *T. vulgare*. Bar: 10 µm

**Fig. 2:**
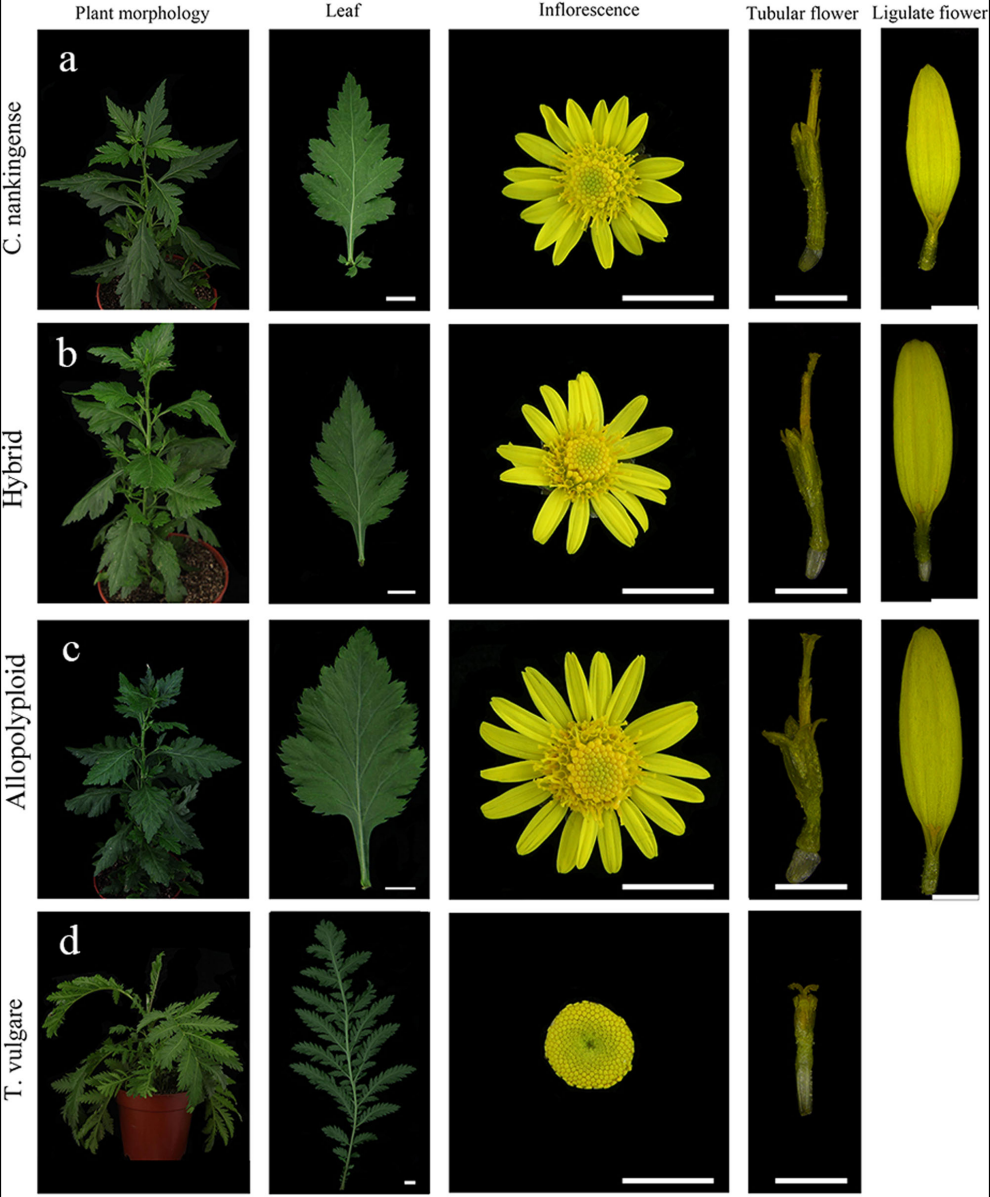
Morphology of materials. **a** *C. nankingense*. **b** Hybrid. **c** Allopolyploid. **d** *T. vulgare*. Leaf bar: 1 cm; inflorescence bar: 1 cm; tubular flower bar: 2 mm; ligulate flower bar: 2 mm

The materials used were maternal parent *C. nankingense*, paternal parent *T. vulgare*, and a *C. nankingense*×*T. vulgare* hybrid, and allopolyploids (Figs. 1 and 2). The three allopolyploids were generated from a chromosome-doubled *C. nankingense*×*T. vulgare* hybrid, and they had similar phenotypes to one another. The materials were maintained by the Chrysanthemum Germplasm Resource Preserving Center, Nanjing Agricultural University, China (32°05′N, 118°8′E, 58 m altitude) and were propagated by cuttings. Plants were grown in a greenhouse (22 °C during the day and a minimum of 15 °C at night; relative humidity of 70–75%; under natural light).

### Genome doubling

Nodal segments from 1-month-old *C. nankingense*×*T. vulgare* hybrid plantlets were immersed in 500 mg/l colchicine for 48 h and then rinsed three times in sterile water and placed on hormone-free MS medium for 1 month. Then, the developed lateral buds were excised and transferred to rooting medium.

### Chromosome counting

Young root tips (1–2 cm) were collected and pretreated in ice water for 20–24 h, fixed in Carnoy’s solution (3:1 ethanol:glacial acetic acid (v/v)), and stored at 4 °C for 24 h. The fixed root tips were squashed under a glass slide in a drop of 45% (v/v) glacial acetic acid. The resulting mitotic chromosome spreads were observed via phase contrast microscopy (Olympus BX41, Tokyo, Japan).

### Morphological trait analysis

A set of hybrid and allopolyploid morphological traits were measured: plant height, leaf length, leaf width and leaf stalk (using the fourth leaf from the apex), inflorescence diameter, ligulate flower quantity, and tubular flower quantity^29,31^. The analyzed measurements were the means of ten replications. The shape of the aboveground parts, leaf shape and flower shape were photographed.

### Sequence-related amplified polymorphism analysis

DNA was extracted from the fourth leaf of three individual plants of the two parental lines, hybrid line and the three allopolyploids using a modified CTAB method^32^. The DNA was used for SRAP profiling as described by Li and Quiros^33^. A total of 63 SRAP primer pairs were used, including 19 forward and 16 reverse primers (Table S1).The pairs were ME-1 combined with EM-1 (abbreviated “ME1+EM1”), ME1+EM7, ME1+EM14, ME1+EM16, ME3+EM1, ME3+EM2, ME3+EM5, ME3+EM9, ME3+EM10, ME3+EM11, ME3+EM17, ME5+EM1, ME5+EM7, ME5+EM8, ME5+EM10, ME6+EM1, ME6+EM2, ME6+EM5. ME6+EM14, ME8+EM14, ME8+EM15, ME9+EM1, ME10+EM1, ME10+EM2, ME10+EM4, ME10+EM7, ME10+EM11, ME10+EM14, ME10+EM15, ME11+EM11, ME11+EM19, ME12+EM2, ME12+EM12, ME12+EM15, ME14+EM2, ME14+EM5, ME15+EM17, ME16+EM2, ME16+EM10, ME16+EM11, ME16+EM14, ME16+EM15, ME16+EM19, ME17+EM1, ME17+EM5, ME17+EM7, ME17+EM9, ME17+EM15, ME18+EM8, ME18+EM10, ME19+EM5, ME19+EM12, ME20+EM2, ME21+EM2, ME21+EM4, ME21+EM19, ME23+EM6, ME24+EM2, ME24+EM4, ME24+EM9, ME24+EM11, ME24+EM15, and ME24+EM16. Each 25 μl reaction mix comprised 15 ng genomic DNA, 2.5 μl of 10x PCR buffer, 1.5 mM MgCl_2_, 0.2 mM dNTPs, and 2 U of Taq polymerase (Takara, Tokyo, Japan). The reactions were first denatured (94 °C/5 min); followed by 5 cycles of 94 °C/1 min, 35 °C/1 min, and 72 °C/2 min; followed by 35 cycles of 94 °C/1 min, 50 °C/1 min, and 72 °C/2 min; with a final extension step of 72 °C/7 min. The SRAP amplicons were electrophoresed with 6% denaturing polyacrylamide gels and visualized via silver staining. Fragments in the size range of 100–500 bp were scored.

### Methylation-sensitive amplification polymorphism analysis

The MSAP procedure was based on the protocol of Wang et al. with minor modifications^30^. A 500 ng genomic DNA sample from each hybrid and allopolyploid plant was digested with either 10 U of *Eco*RI (New England Biolabs) and 20 U of *Hpa*II (New England Biolabs) or 10 U of *Eco*RI and 10 U of *Msp*I (New England Biolabs). The products were ligated to 5 pmol *Eco*RI and 50 pmol of the *Hpa*II*/Msp*I adaptors using 4 U of T4 DNA ligase in a reaction. The pre-selection amplification reaction contained 5 μl of the ligation product, 0.2 μM *Eco*RI and 0.2 μM *Hpa*II*/Msp*I non-selective primers in a 25 μl reaction. The pre-amplification products were diluted at 1:30 with sterile ultra-pure H_2_O to provide the template for the subsequent selective amplification. Twenty primer combinations of *Eco*RI selective primer #1 and *Hpa*II*/Msp*I selective primer #2 (abbreviated E1+HM2), E1+HM6, E2+HM6, E2+HM8, E3+HM1, E3+HM5, E4+HM2, E4+HM3, E4+HM6, E4+HM7, E4+HM8, E5+HM2, E5+HM3, E5+HM4, E6+HM1, E6+HM6, E7+HM6, E7+HM8, E8+HM7, and E8+HM8 were used in the selective amplification reaction (Table S2). The MSAP amplicons were electrophoresed through 8% nondenaturing polyacrylamide gels and visualized by silver staining. For the statistical analysis, fragments in the size range of 100–500 bp were scored.

### RNA extraction and RNA-Seq analysis

The fourth leaf of three individual plants of the two parental lines *C. nankingense* (Jhn) and *T. vulgare* (Jh); the hybrid line (JJ); and the allopolyploid line (JJD) were harvested and snap frozen in liquid nitrogen until RNA extraction. Total RNA was extracted using RNAiso reagent (Takara, Japan) according to the manufacturer’s recommendations. The integrity and quality of the total RNA were verified using a 2100 Bioanalyzer RNA Nano chip (Agilent, Santa Clara, CA, USA). The concentration was measured with an ND-430 1000 spectrophotometer (NanoDrop, Wilmington, DE). The RNA was stored at −80 °C for subsequent use.

The mRNA of each library was sequenced on an Illumina HiSeq^TM^ 4000 platform located at the Beijing Genomics Institute (Shenzhen, China; http://www.genomics.cn/index). The NT (ftp://ftp.ncbi.nlm.nih.gov/blast/db), NR (ftp://ftp.ncbi.nlm.nih.gov/blast/db), COG (http://www.ncbi.nlm.nih.gov/COG), KEGG (http://www.genome.jp/kegg), and Swiss-Prot (http://ftp.ebi.ac.uk/pub/databases/swissprot) databases were used for blast search and annotation^34^. Blast2GO (v2.5.0) was used to obtain the GO (http://geneontology.org) annotation^35^, and InterProScan5 (v5.11–51.0) was used to obtain the InterPro (http://www.ebi.ac.uk/interpro) annotation^36^. Blast similarity searches were performed for pairwise comparisons of all four libraries (JJ-VS-Jhn, JJ-VS-Jh, JJD-VS-Jhn, JJD-VS-Jh, Jhn-VS-Jh, and JJD-VS-JJ). Orthologous and homoeologous genes were both standardized by the following criteria: *E*-value ≤9E^−100^, alignment length ≥200 bp, and identity ≥90%. Fragments per kilo base per million (FPKM) was used to estimate the expression levels of genes and to compare the differences of gene expression among samples. Differentially expressed genes (DEGs) were identified through an algorithm developed by Audic and Claverie^37^. The criterion applied was |log_2_Ratio| ≥ 1.0. The *in silico* midparent value (MPV) was calculated by averaging the values of the parents to mimic parental additivity in the hybrids and the allopolyploids.

### Quantitative real-time PCR (qRT-PCR) validation of DEGs

Total RNA was extracted from the fourth leaf of three individual plants of the two parental lines and the hybrid line using RNAiso reagent according to the manufacturer’s instructions. Three biological replicates and three technical replicates were used for qRT-PCR analysis. Primers were designed using Primer 5.0 software (sequences given in Table S3). The *C. nankingense* *EF1α* gene was used as the reference. The PCR cycles consisted of an initial denaturation (95 °C/2 min) followed by 40 cycles of 95 °C/15 s, 55 °C/15 s, and 72 °C/20 s. Relative expression levels were calculated using the 2^−△△CT^ method.

## Results

### Chromosome number and phenotype analysis

A series of allopolyploids were generated through colchicine treatment of a *C. nankingense*×*T. vulgare* hybrid. We investigated the ploidy of the seedlings using chromosome counts. The somatic chromosome number of *C. nankingense* was 2*n* = 18 (Fig. 1a), that of *T. vulgare* was 2*n* = 18 (Fig. 1d) and that of the *C. nankingense*×*T. vulgare* hybrid was 2*n* = 18 (Fig. 1b). As expected, the allopolyploid somatic chromosome number was 36 (Fig. 1c). Three plants out of 35 novel seedlings generated with the colchicine treatment were putative allopolyploids and had similar phenotypes.

The mature hybrid and allopolyploid plants were characterized morphologically. *T. vulgare* has no ligulate flowers (Fig. 2). *C. nankingense* appeared to be morphologically dominant over *T. vulgare* in the hybrid and allopolyploids. Leaf length and width were significantly greater in the allopolyploids than in the hybrid (Fig. 2; Table 1). Inflorescence diameter and floret (both ligulate and tubular) size were larger in the allopolyploids than in the hybrid. Although tubular flower quantity increased, there was no significant difference in ligulate flower quantity between the hybrid and allopolyploid plants. Flowering time showed no difference between the hybrid and allopolyploids.

**Table 1:** Phenotypic comparison between the hybrid and allopolyploids

Traits	Hybrid	Allopolyploids
Plant height (cm)	62.3 ± 0.33b	82.7 ± 0.67a
Leaf length (cm)	5.29 ± 0.76b	7.29 ± 0.21a
Leaf width (cm)	3.19 ± 0.04b	5.32 ± 0.28a
Leaf stalk (cm)	1.14 ± 0.09b	1.33 ± 0.02a
Inflorescence diameter (cm)	2.03 ± 0.06b	2.57 ± 0.13a
Ligulate flower quantity	17.7 ± 0.33a	18.3 ± 0.33a
Tubular flower quantity	103.3 ± 2.73b	117.1 ± 1.53a

### Genomic changes in the hybrid and allopolyploids

Sixty-three SRAP primer pairs amplified 525 fragments from *C. nankingense*, 480 fragments from *T. vulgare* and 630 fragments from the hybrid. Among the 630 fragments from the hybrid, 268 (42.5%) were present in the profiles of both parents (Fig. S1a; Table 2), 212 (33.7%) were inherited from *C. nankingense* (Fig. S1b; Table 2) and 146 (23.2%) were from *T. vulgare* (Fig. S1c; Table 2). Four novel fragments were detected in the hybrid, and all of them were transmitted to the allopolyploids, except allopolyploid 3 (Fig. S1f; Table 2). Allopolyploids 1/2/3 amplified 634/636/631 fragments, of which 269/271/269 were present in both parents’ profiles, 215/213/214 were inherited from *C. nankingense*, 146/148/146 were from *T. vulgare* and 4/4/2 were novel fragments.

**Table 2:** Fragment types in the SRAP analysis of the hybrid and allopolyploids

Fragments type	Hybrid		Allopolyploid 1/2/3	
	Number	Percentage (%)	Number	Percentage (%)
Common fragments	268	42.5	269/271/269	42.4/42.6/42.6
Maternal-special fragments	212	33.7	215/213/214	33.9/33.5/33.9
Paternal-special fragments	146	23.2	146/148/146	23.1/23.3/23.2
Novel fragments	4	0.6	4/4/2	0.6/0.6/0.3
Total fragments	630	100	634/636/631	100/100/100

To explore the effects resulting from hybridization or polyploidization, the genetic alterations were further divided into three types according to the stage at which the alterations occurred. The hybridization-only (H-only) type refers to the alterations that initially occurred in the hybrid and were transmitted to the allopolyploids; this type indicated that alterations were induced by hybridization. The polyploidization-only (P-only) type refers to the alterations that initially occurred in the allopolyploids but not in the hybrid, indicating that alterations were induced by polyploidization. The hybridization–polyploidization (H–P) type refers to the alterations that initially occurred in the hybrid but were later recovered in the allopolyploids. This type indicated that the corresponding sites were affected by both hybridization and polyploidization. We found 84/84/85 fragments (84%/85.7%/85% of total alterations) altered in both the hybrid and allopolyploids (H-only type), of which 23/24/24 fragments from *C. nankingense* disappeared and 46/46/47 fragments from *T. vulgare* disappeared (Table 3). There were 6/4/6 (6%/4.1%/6%) fragments that disappeared in the allopolyploids but were present in the hybrid (P-only type). There were 10/10/9 (10%/10.2%/9%) fragments that disappeared in the hybrid but were recovered in the allopolyploids (H–P type).

**Table 3:** SRAP fragments affected by either hybridization or polyploidization

	*C. nankingense*	*T. vulgare*	Hybrid	Allopolyploid	Number of variable fragments of allopolyploid 1/2/3	Total
H-only type^a^	+	−	−	−	23/24/24	84/84/85
	−	+	−	−	46/46/47	
	+	+	−	−	15/14/14	
P-only type^b^	+	−	+	−	2/3/2	6/4/6
	−	+	+	−	3/1/2	
	+	+	+	−	1/0/2	
H–P type^c^	+	−	−	+	5/4/4	10/10/9
	−	+	−	+	3/3/2	
	+	+	−	+	2/3/3	
Subtotal					100/98/100	

+ Fragment present, − fragment absent

^a^H-only type refers to the alterations initially occurred in the hybrid and transmitted to the allopolyploids

^b^P-only type refers to the alterations initially occurred in the allopolyploids but not in the hybrid

^c^H–P type refers to the alterations initially occurred in the hybrid but later recovered in the allopolyploids

### Epigenetic changes between the hybrid and allopolyploids

Fragments sharing both digestions indicated that the corresponding restriction sites CCGG were not methylated (type I, non-methylated). A fragment detected in only the *Eco*RI + *Msp*I (M lane) digestion contained methylation of the internal cytosines on both strands (type II, fully methylated). Fragments appearing in only the *Eco*RI + *Hpa*II (H lane) digestions were attributed to hemi-methylated fragments (type III, hemi-methylated). Type IV fragments were present in either the H or M lane of the hybrid but were absent from both H and M lanes in allopolyploids, indicating increased methylation or were absent from both H and M lanes in the hybrid but present in either H and M lane in the allopolyploids, implying decreased methylation.

Twenty MSAP primer pairs were used to detect changes in methylation status. The hybrid amplified 202 type I, 127 type II, and 116 type III fragments, and the proportion of methylated fragments was 54.6%; the allopolyploids profile included 198/198/201 type I, 134/132/135 type II, and 122/122/119 type III fragments, and the proportion of methylated fragments was 56.4%/56.2%/55.8% (Table 4). The number of fragments exhibiting a changed methylation state between the hybrid and allopolyploids was 77.2 on average, of which 51.5 showed an increase in methylation and 25.7 exhibited a decrease in methylation. There were 2.8 sites that shifted from type IV to type I, 3.5 from type IV to type III, 4.3 from type IV to type II, 9.3 from type II to type I, and 5.8 from type III to type I (decreased methylation); 3.3 sites shifted from type I to type IV, 11.8 from type III to type IV, 9.3 from type II to type IV, 16.8 from type I to type II, and 10.3 from type I to type III (increased methylation) (Table 5).

**Table 4:** Levels of cytosine methylation detected in the hybrid and allopolyploids

Plant lines	Total sites	Non-methylated	Methylated		
		Type I	Total (II+III)	Type II	Type III
Hybrid	445	202 (45.4%)	243 (54.6%)	127 (28.5%)	116 (26.1%)
Allopolyploid 1	454	198 (43.6%)	256 (56.4%)	134 (29.5%)	122 (26.9%)
Allopolyploid 2	452	198 (43.8%)	254 (56.2%)	132 (29.3%)	122 (26.9%)
Allopolyploid 3	455	201(44.2%)	254 (55.8%)	135 (29.7%)	119 (26.1%)

**Table 5:** MSAP fragments affected by polyploidization

Fragment type		Fragment display pattern in MSAP gel				Number of sites	Status
Hy	Al	Hy H lane	Hy M lane	Al H lane	Al M lane		
Type IV	Type I	−	−	+	+	2.8 ± 0.5	↓
Type I	Type IV	+	+	−	−	3.3 ± 0.2	↑
Type IV	Type III	−	−	+	−	3.5 ± 0.3	↓
Type III	Type IV	+	−	−	−	11.8 ± 0.5	↑
Type IV	Type II	−	−	−	+	4.3 ± 0.2	↓
Type II	Type IV	−	+	−	−	9.3 ± 0.3	↑
Type II	Type I	−	+	+	+	9.3 ± 0.5	↓
Type I	Type II	+	+	−	+	16.8 ± 0.5	↑
Type III	Type I	+	−	+	+	5.8 ± 0.3	↓
Type I	Type III	+	+	+	−	10.3 ± 0.5	↑

Hy: hybrid, Al: allopolyploid, ↓: decreased methylation, ↑: increased methylation

### Genes expressed in the hybrid /allopolyploid and parents

**Fig. 3:**
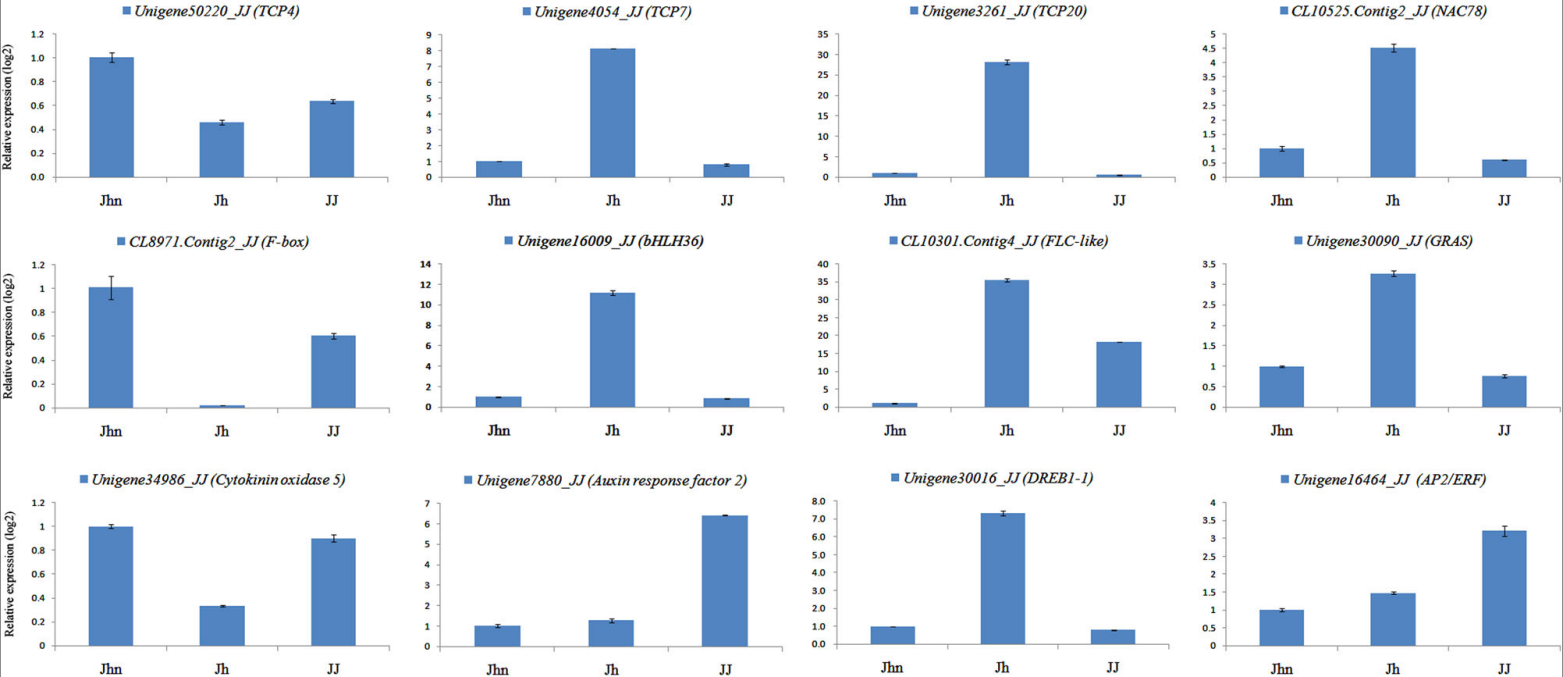
Verification of RNA-Seq results by qPCR between the hybrid and its parents. Jhn: *C. nankingense*, Jh: *T. vulgare*, JJ: hybrid, JJD: allopolyploid

Using the Illumina HiSeq^TM^ 4000 platform, a total of 44.15, 45.28, 45.24, and 44.34 Mb clean reads were generated from the libraries of *C. nankingense* (Jhn), *T. vulgare* (Jh), the hybrid (JJ), and the allopolyploid (JJD), respectively; in total, 73,990, 84,846, 81,603, and 81,107 unigenes, respectively, were found in each library (Table 6). The range of unigene length was from 200 to 15,730 bp (means were 823, 939, 805, and 831 bp in each library, respectively) (Figure S2). We verified the expression patterns of the genes that were significantly expressed in the samples by qRT-PCR. The expression trends were generally consistent with the transcript abundances estimated from the RNA-Seq data, although the selected genes showed different fold-change values (Fig. 3).

**Table 6:** Summary of read mapping

Sample	Jhn	Jh	JJ	JJD
Total raw reads (Mb)	53.9	55.53	55.53	55.53
Total clean reads (Mb)	44.15	45.28	45.24	44.34
Total clean bases (Gb)	6.62	6.79	6.79	6.65
Clean reads ratio (%)	81.48	79.85	81.54	81.92
Clean reads Q20 (%)	98.5	98.47	98.52	98.42
Clean reads Q30 (%)	95	94.94	95.1	94.82
Total number of unigenes	73990	84846	81603	81107
Total length of unigenes (bp)	60,942,576	79,707,000	65,716,489	67,406,068
Mean length of unigenes (bp)	823	939	805	831
GC percentage (%)	40.13	39.53	39.82	39.94

Jhn: *C. nankingense*, Jh: *T. vulgare,* JJ: hybrid, JJD: allopolyploid

**Fig. 4:**
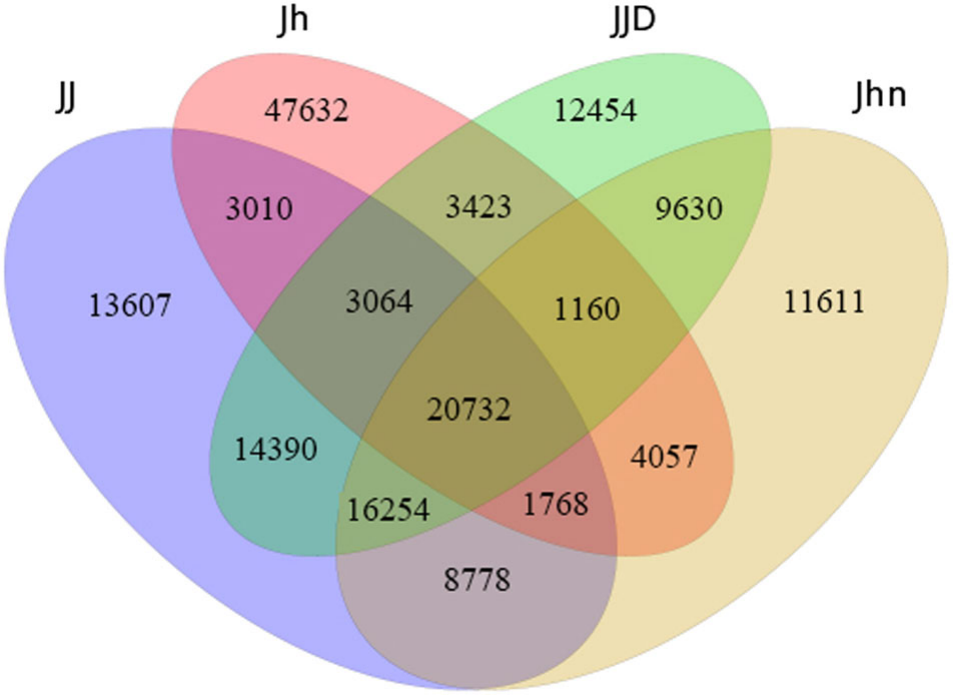
The number of genes detected in the libraries of *C. nankingense*, *T. vulgare*, the hybrid, and the allopolyploid. Jhn: *C. nankingense*, Jh: *T. vulgare*, JJ: hybrid, JJD: allopolyploid

In the comparison of the genes expressed between the hybrid and its parents (Fig. 4), a total of 22,500 genes were shared by the hybrid and its parents, 5217 were co-expressed in *C. nankingense* and *T. vulgare*, 25,032 were specifically co-expressed in *C. nankingense* and the hybrid, and 6074 were specifically co-expressed in *T. vulgare* and the hybrid. A total of 21,241 (*C. nankingense*) and 51,055 (*T. vulgare*) genes were specifically expressed, and for 27,997 hybrid novel genes, the exact source could not be detected.

In the comparison of the genes expressed between the allopolyploid and its parents (Fig. 4), there were 21,892 genes shared by the allopolyploid and its parents, 5825 were co-expressed in *C. nankingense* and *T. vulgare*, 25,884 were specifically co-expressed in *C. nankingense* and the allopolyploid, and 6487 were specifically co-expressed in *T. vulgare* and the allopolyploid. There were 20,389 (*C. nankingense*) and 50,642 (*T. vulgare*) genes specifically expressed, and for 26,844 allopolyploid novel genes, the exact source could not be detected.

A total of 81,603 genes were expressed in the hybrid, of which 22,500 (27.6%) genes were co-expressed in the hybrid and both parents, 25,032 (30.7%) were co-expressed in the hybrid and *C. nankingense*, and 6074 (7.4%) were co-expressed in the hybrid and *T. vulgare*. In the allopolyploid, 81,107 genes were detected, of which 21,892 (27.0%), 25,884 (31.9%), and 6487 (7.9%) genes were co-expressed in the allopolyploid and both parents, the allopolyploid and *C. nankingense*, and the allopolyploid and *T. vulgare*, respectively.

### DEGs and functional analysis

**Fig. 5:**
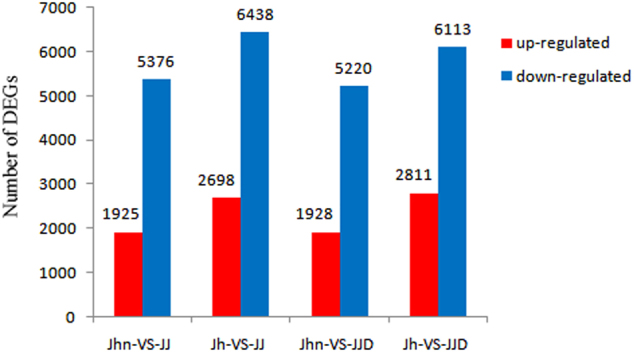
Differentially expressed genes detected between the hybrid and its parents and between the allopolyploid and its parents. Jhn-VS-JJ: comparison between Jhn and JJ, Jh-VS-JJ: comparison between Jh and JJ, Jhn-VS-JJD: comparison between Jhn and JJD, Jh-VS-JJD: comparison between Jh and JJD. Jhn: *C. nankingense*, Jh: *T. vulgare*, JJ: hybrid, JJD: allopolyploid

Among the 22,500 genes co-expressed in the hybrid and its parents, 11,379 genes showed at least a two-fold differential expression level. Compared with *C. nankingense*, the hybrid had 1925 up-regulated genes and 5376 down-regulated genes (Fig. 5; Table S4). Comparing the hybrid and *T. vulgare*, 2698 genes were up-regulated and 6438 genes were down-regulated in the hybrid (Fig. 5; Table S4). There were more DEGs between the hybrid and *T. vulgare* than between the hybrid and *C. nankingense* (9136 vs. 7301).

Among the 21,892 genes co-expressed in the allopolyploid and its parents, 11,207 genes showed at least a two-fold change in gene expression level. Between the allopolyploid and *C. nankingense*, 1928 genes were up-regulated and 5220 genes were down-regulated (Fig. 5; Table S4), whereas 2811 and 6113 genes were up-regulated and down-regulated, respectively, when comparing the allopolyploid with *T. vulgare* (Fig. 5; Table S4). The DEG number between the allopolyploid and *T. vulgare* was larger than that between the allopolyploid and *C. nankingense* (8924 vs. 7148).

**Fig. 6:**
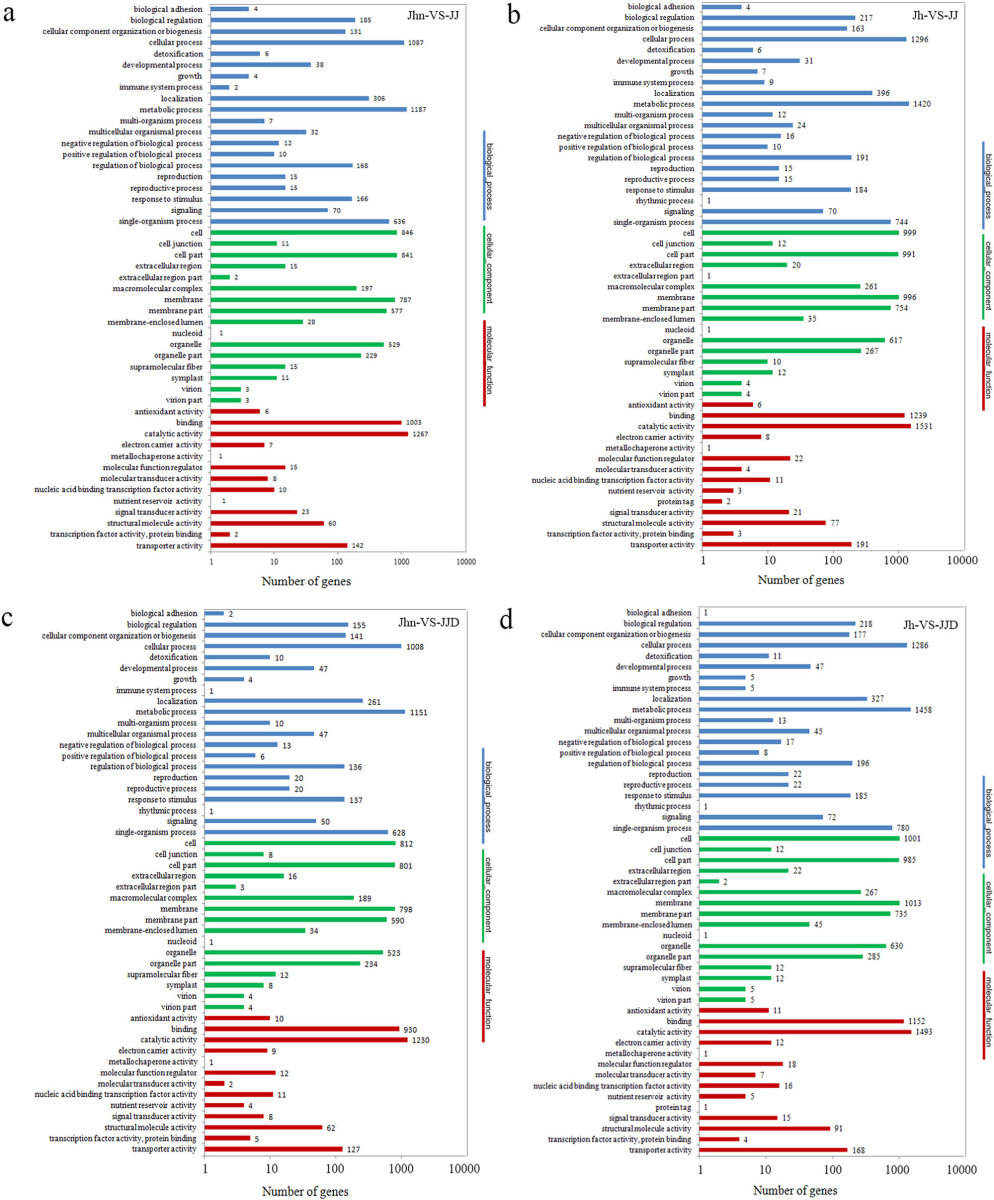
Gene ontology functional classification of differentially expressed genes. **(a)** Comparison between Jhn and JJ (Jhn-VS-JJ), **(b)** comparison between Jh and JJ (Jh-VS-JJ), **(c)** comparison between Jhn and JJD (Jhn-VS-JJD), **(d)** comparison between Jh and JJD (Jh-VS-JJD). DEGs were annotated in three categories: biological process (blue), cellular component (green), and molecular function (red). Jhn: *C. nankingense*, Jh: *T. vulgare*, JJ: hybrid, JJD: allopolyploid

We categorized the DEGs according to the secondary classification of GO terms. In the Jhn-VS-JJ, 2567 of the 7301 DEGs could be assigned a GO term; the numbers for the other comparisons were as follows: Jh-VS-JJ, 3126/9136; Jhn-VS-JJD, 2469/7148; and Jh-VS-JJD, 3075/8924. Genes belonged to three main GO classification categories: biological process, cellular component and molecular function (Fig. 6; Table S5). The terms metabolic process and cellular process were dominant in the biological process category. The major classes of the cellular components category were the terms cell, cell part, and membrane. The terms catalytic activity and binding were dominant in the molecular function category.

DEGs were mapped to KEGG pathways. For Jhn-VS-JJ, 3041 DEGs mapped to 132 pathways; for Jh-VS-JJ, 3898 DEGs mapped to 132 pathways; for Jhn-VS-JJD, 3034 DEGs mapped to 132 pathways; and for Jh-VS-JJD, 3821 DEGs mapped to 131 pathways (Table S6). The major enrichments among metabolic pathways were the biosynthesis of secondary metabolites, RNA transport, carbon metabolism, biosynthesis of amino acids, spliceosome, starch and sucrose metabolism, endocytosis, plant-pathogen interaction, protein processing in endoplasmic reticulum, and plant hormone signal transduction.

### Clustering of DEGs

Through hierarchical clustering, we analyzed DEG associations based on the differences and correlations among their expression patterns. We classified 4854 significant DEGs between the hybrid and *C. nankingense* and between the hybrid and *T. vulgare* into four expression patterns using two-dimensional hierarchical clustering (Table S7). Cluster 1, the most abundant cluster, contained 3787 genes that were down-regulated in the hybrid when compared with its parents. Cluster 2 had 179 genes that were down-regulated in the hybrid when compared with its maternal parent, *C. nankingense*, and were up-regulated when compared with its paternal parent, *T. vulgare*. Cluster 3 included 270 genes that were up-regulated in the hybrid when compared with *C. nankingense* and down-regulated when compared with *T. vulgare*. The second most abundant cluster, cluster 4, had 618 genes that were up-regulated in the hybrid when compared with its parents. We also categorized 4665 genes with significant differential expression between the allopolyploid and *C. nankingense* and between the allopolyploid and *T. vulgare* into four clusters (Table S8). Cluster 1 contained 3611 genes, cluster 2 contained 205 genes, cluster 3 contained 219 genes, and cluster 4 contained 630 genes; cluster 1 was the most abundant cluster.

A total of 11,379 DEGs showed differential expression between the hybrid and *C. nankingense* or between the hybrid and *T. vulgare*, and we divided them into four groups (Table S9). Group 1 had 6392 genes, group 2 had 1244, group 3 had 1304, and group 4 had 2439; group 1 was the most abundant group. Genes in groups 1, 2, 3, and 4 had the same expression profiles as genes in clusters 1, 2, 3, and 4. We also classified 11,207 DEGs between the allopolyploid and *C. nankingense* or between the allopolyploid and *T. vulgare* into four groups (Table S10). There were 6171, 1222, 1229, and 2585 genes in groups 1, 2, 3, and 4, respectively, and group 1 was the most abundant group.

### Non-additive genes expressed in the hybrid and the allopolyploid

**Fig. 7:**
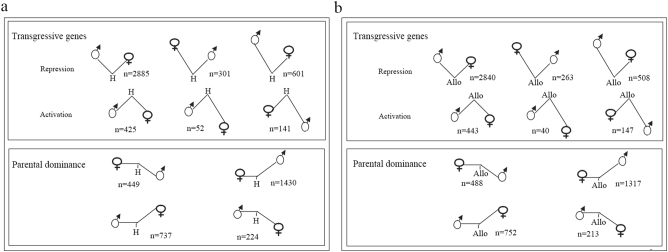
Non-additive gene expression patterns (and number of non-additive genes in each condition) in the hybrid (H), maternal parent *C. nankingense* and paternal parent *T. vulgare*, and in the allopolyploid (Allo), *C. nankingense* and *T. vulgare*. **a** Expression patterns in the hybrid (H), *C. nankingense*, and *T. vulgare*. **b** Expression patterns in the allopolyploid (Allo), *C. nankingense*, and *T. vulgare*. Non-additive genes displayed transgressive patterns (repression or activation) and the parental dominance pattern. *n* represents the corresponding number of genes in each condition

Genes showing at least a two-fold change in expression level between the hybrid/allopolyploid and the midparent value (MPV) were considered non-additive genes; all others were considered additive genes. According to the analytical method described by Chelaifa et al.^10^, we distinguished the non-additive genes that displayed transgressive patterns (overexpressed or underexpressed compared with the parents) from those showing parental dominance. In the hybrid, there were 13,811 (65.6%) additive genes and 7245 (34.4%) non-additive genes. Among these non-additive genes (Fig. 7a), a greater number of genes exhibited transgressive expression (*n* = 4405) than paternal expression dominance (*n* = 2840) and thus appeared to be more important, especially for the repression pattern (*n* = 3787). The number of genes that exhibited the maternal expression dominance pattern (*n* = 1879) was greater than the number displaying the paternal expression dominance pattern (*n* = 961). In the allopolyploid, there were 13,610 (66%) additive genes and 7011 (34%) non-additive genes. Among these non-additive genes (Fig. 7b), transgressive expression patterns appeared to be dominant (*n* = 4241), especially the repression pattern (*n* = 3611). The number of genes that showed the maternal expression dominance pattern (*n* = 1805) was greater than the number displaying the paternal expression dominance pattern (*n* = 965).

To study the differences in functional category distribution between the additive and non-additive genes, we categorized them by secondary GO terms. The genes belonged to 21, 16, and 13 functional groups in the three main GO categories: biological process, cellular component, and molecular function (Figure S3; Table S11). In both the hybrid and the allopolyploid, the terms biological regulation, cellular component organization or biogenesis, cellular process, localization, metabolic process, regulation of biological process, response to stimulus, and single-organism process showed significant differences in the numbers of additive and non-additive genes and were enriched in additive genes in the biological process category. In the cellular components category, the numbers of additive and non-additive genes differed significantly for the functional terms cell, cell part, macromolecular complex, membrane, membrane part, organelle, and organelle part and were enriched in additive genes. In the molecular function category, the terms binding, catalytic activity, structural molecule activity, and transporter activity had significantly more additive genes than non-additive genes.

### DEGs related to methyltransferase and methylase genes

We also detected the differentially expressed methyltransferase and methylase genes between the hybrid/allopolyploid and its parents (Table S12). There were 108 methyltransferase genes significantly differentially expressed between the hybrid and its parents. Between the hybrid and *C. nankingense*, 74 methyltransferase genes were differentially expressed in the hybrid, 14 were up-regulated and 60 were down-regulated. Relative to *T. vulgare*, in the hybrid, 20 methyltransferase genes were up-regulated and 66 were down-regulated. Sixteen methylase genes were identified as significantly differentially expressed between the hybrid and its parents. One methylase gene was up-regulated and 11 were down-regulated between the hybrid and *C. nankingense*; one was up-regulated and 13 were down-regulated between the hybrid and *T. vulgare*. We detected 130 significantly differentially expressed methyltransferase genes between the allopolyploid and its parents. There were 14 up-regulated and 74 down-regulated methyltransferase genes between the allopolyploid and *C. nankingense* and 28 up-regulated and 73 down-regulated between the allopolyploid and *T. vulgare*. Twelve methylase genes were identified as significantly differentially expressed between the allopolyploid and its parents. Nine were down-regulated between the allopolyploid and *C. nankingense*, and nine were down-regulated between the allopolyploid and *T. vulgare*.

## Discussion

### Genomic changes under intergeneric hybridization and polyploidization

Plants tolerate hybridization and polyploidization well. Hybrids express certain morphologies clearly inherited from one of the parents and some *de novo* morphologies that are apparently not inherited from either^38,39^. Nascent F1 hybrids often experience subtle fragment variations that involve two major types of genetic change: the loss of parental fragments and the gain of novel fragments^30^. Synthetic allopolyploids underwent DNA sequence elimination between wheat and its progenitors^19^ and between resynthesized *Brassica napus* and its parents, *Brassica oleracea* and *Brassica rapa*^20^. In the present study, SRAP profiling showed that the contributions of the parents to the hybrid were 33.7% (*C. nankingense*) and 23.2% (*T. vulgare*). Similarly, 34% female-specific fragments and 22.1% male-specific fragments were detected in the *C. nankingense*×*T. vulgare* hybrid using AFLP profiling^30^. The hybrid failed to inherit a number of parental fragments; 5.3% (28/525) maternal fragments and 10.2% (49/480) paternal fragments were not present in the hybrid. These rates are all higher than those reported for the newly synthesized wheat allopolyploid^4,40^. If point mutations remove or create a restriction site, new fragments may form. Here, four novel fragments were detected, and all of them were transmitted to the allopolyploids except for allopolyploid 3.

Hybridization is the major force driving genomic changes^40^. In the present study, we found that 84/84/85 fragment alterations (84%/85.7%/85% of total alterations) were induced by hybridization, 6/4/6 (6%/4.1%/6%) fragment alterations were induced by polyploidization, and 10/10/9 (10%/10.2%/9%) fragment alterations were induced by both hybridization and polyploidization. Our results indicate that hybridization is a major force in newly formed allopolyploid genomic changes. The formation of allopolyploids may not simply be the sum of hybridization and polyploidization changes but appears also to involve an interaction between these factors.

There were 84/84/85 fragments altered in both the hybrid and allopolyploid; 46/46/47 disappeared from *T. vulgare*, whereas only 23/24/24 disappeared from *C. nankingense*. The maternal parent, *C. nankingense*, was dominant in the hybrid and allopolyploid. This phenomenon was also detected in *Brassica*, and 20 bands changed in both the hybrids and allohexaploids, of which 5 and 12 specific fragments from *B. carinata* and *B. rapa* disappeared, respectively^40^. The mechanisms for progenitor-biased alterations have yet to be determined. It was reported that the cytoplasmic background might affect genetic changes in resynthesized *Brassica*^41^.

### Epigenetic changes under polyploidization

Genomic changes in hybrid or allopolyploid individuals are not the only potential driver of polyploidization; epigenetic changes also have an important role. Epigenetic changes occurred in the early stage of synthetic wheat^18^, *Brassica*^42^, *Arabidopsis*^43^, and *Senecio*^44^ allopolyploids and were maintained after polyploidization. However, polyploidization also results in the reversion of hybridization-induced methylation alterations and novel methylation changes in the allopolyploids^18,43,44^. A previous study revealed that the methylation level of the *C. nankingense*×*T. vulgare* hybrid was reduced by its wide hybridization;^30^ in this study, we found that the level was somewhat restored after polyploidization, and the number of fragments with increased DNA methylation in the allopolyploid was twice (51.5/25.7) that with decreased methylation (Table 5). A study of cytosine methylation in a newly synthesized allopolyploid in *Cucumis* revealed that cytosine methylation changes showed an increase of twice the level of decrease observed between the reciprocal F1 hybrids and the allopolyploid^45^. Furthermore, most full-CG methylation alterations that occurred in the hybrids were recovered after polyploidization, whereas hemi-CCG methylation alterations were relatively stable when transferred from hybrids to allopolyploids (Table 4). This finding confirms that polyploidization results in the reversion of hybridization-induced DNA methylation alterations and novel methylation alterations in the allopolyploids^40^.

DNA methylation has an important role in the transcriptional changes in hybrids and allopolyploids. Methylation pattern alterations affected both low-copy DNA and repetitive DNA sequences^18^. Major methylation changes were detected in the vicinity of transposable elements following hybridization^46^. Changes to the methylation state in triploids and allohexaploid derivatives mirrored non-additive gene expression patterns^44^. In this study, we detected 108 methyltransferase genes and 16 methylase genes that were significantly differentially expressed between the hybrid and its parents, and 130 methyltransferase genes and twelve methylase genes were detected between the allopolyploid and its parents. Gene methylation leads to gene inactivation; thus, the up-regulation of methyltransferase and methylase genes may result in the down-regulation of some DEGs, and the down-regulation of methylation-related genes may lead to the up-regulation of some DEGs in the hybrid/allopolyploid compared with its parents. Whether these methylation-related genes changes are associated with the different expression levels of the DEGs in the hybrid/allopolyploid needs to be verified.

### Parental-biased gene expression under intergeneric hybridization and polyploidization

The respective effects of hybridization and polyploidization on transcriptomic changes have been investigated in many allopolyploid systems^11,17,40,42^. With the increasing availability of RNA-Seq technology, this technique has been used for transcriptomic analysis in a number of plant species^13,47–51^.

The genes co-expressed in *C. nankingense* and the hybrid were abundant than the genes co-expressed in *T. vulgare* and the hybrid (15.7 vs. 3.8%). Similarly, the genes co-expressed in *C. nankingense* and the allopolyploid were more abundant than the genes co-expressed in *T. vulgare* and the allopolyploid (16.4 vs. 4.1%). The DEGs between the hybrid and *C. nankingense* accounted for 32.4%, whereas between the hybrid and *T. vulgare*, the percentage was 40.6%. The DEGs between the allopolyploid and *C. nankingense* accounted for 32.6%, whereas between the allopolyploid and *T. vulgare*, the percentage was 40.8%. These findings suggested directional gene expression changes deviating from the paternal parent in the hybrid and the allopolyploid.

Xu et al.^42^ reported the difference was greater between the *Brassica napus* allopolyploid and its paternal parent than between this allopolyploid and its maternal parent. The level of expression changes between a hybrid and its paternal parent, *Spartina maritime*, reached 11.4%, which was equivalent to the proportion of DEGs between the parental species, *S. maritima* and *S. alterniflora*, whereas changes between the hybrid and its maternal parent, *S. alterniflora*, were 2.9%^10^. A study of the trigenomic allohexaploid *Brassica carinata*×*Brassica rapa* showed larger transcriptomic differences between triploid hybrids and the paternal parent^40^. Zhao et al.^13^ detected more DEGs with a larger difference in expression between the *Brassica* hexaploid and its paternal parent, *B. rapa*, than between this hexaploid and its maternal parent, *B. carinata*, and noted directional gene expression changes deviating from the paternal parent. The differences in gene expression between *Populus* allotriploids and the male parent were more significant than those between the allotriploids and the female parent^14^. The explanation for paternal-biased changes in hybrids and allopolyploids is cytoplasmic and maternal effects.

The genes co-expressed in *C. nankingense* and the hybrid showed no greater difference than did the genes co-expressed in *C. nankingense* and the allopolyploid (15.7 vs. 16.4%); *T. vulgare* and the hybrid also showed no larger differences than those in *T. vulgare* and the allopolyploid (3.8 vs. 4.1%). The DEG percentage between the hybrid and *C. nankingense* was 32.4%, and that between the allopolyploid and *C. nankingense* was 32.6%. The DEG percentage between the hybrid and *T. vulgare* was 40.6%, and that between the allopolyploid and *T. vulgare* was 40.8%. The percentage of co-expressed genes and DEGs between the allopolyploid and its parents had the same profile as that between the hybrid and its parents; these findings indicated that the hybridization triggered the majority of the transcriptomic changes.

Studies have demonstrated that polyploidization can influence transcriptomic changes in allopolyploids^12,13,42,52,53^. Further studies have demonstrated that hybridization is principally responsible for transcriptomic changes and that polyploidization affects transcriptomic changes in a manner distinct from hybridization. The majority of protein expression differences in synthesized *B. napus* were found in the F1 hybrids, whereas few variations were associated with genome doubling^54^. With at least 75% of the transcriptomic alterations initiated in the triploid hybrids, it was confirmed that hybridization triggered the majority of the alterations^40^.

### Non-additive gene expression under intergeneric hybridization and polyploidization

Hybrid- or allopolyploid-triggered incompatibilities can be overcome by gene expression changes^55^. A high level of gene expression changes in a non-additive pattern may occur in hybrids derived from distantly related species; these changes provide the molecular bases of hybrid vigor^56^ and of novel changes in the allopolyploid^57^. Although many of the observed gene expression changes in the hybrids were non-additive, the differences in expression level observed in the hybrids were not simply the result of a mixture of parental gene expression levels^58,59^. In this study, among the non-additive genes, transgressive expression patterns appeared to be dominant, especially the repression expression pattern. Maternal expression dominance was more important than paternal expression dominance in both the hybrid (1879 vs. 961) and allopolyploid (1805 vs. 965). The percentage of non-additive genes showed no marked difference between the allopolyploid and the hybrid (34 vs. 34.4%). This finding indicated that the significant changes in non-additive gene regulation observed in the allopolyploid may be induced by intergeneric hybridization. This interpretation is consistent with the results of Wang et al.^26^, who showed that the non-additive gene regulation observed in allopolyploids largely depended on the expression divergence between *A. thaliana* and *A. arenosa* and that the marked changes were induced by interspecific hybridization. These findings also revealed that deviation from parental additivity was most important following hybridization and was accompanied by maternal dominance and transgressive patterns; however, maternal dominance was reduced and transgressive patterns were increased in allopolyploid *S. anglica*^10^.

## Conclusions

Through colchicine treatment, we obtained three putative allopolyploids. Phenotypic analysis between the allopolyploids and the hybrid was performed. Polyploidization resulted in the reversion of hybridization-induced DNA methylation alterations and novel methylation alterations in the allopolyploids. Genomic alterations mainly involved the loss of parental fragments and the gaining of novel fragments. The maternal parent, *C. nankingense*, was dominant in the hybrid and allopolyploids. Most DEGs showed down-regulation in the hybrid/allopolyploid when compared with the parents. Among the non-additive genes, transgressive patterns appeared to be dominant, specifically the repression pattern. Many methyltransferases and methylation genes showed differential expression between the hybrid/allopolyploid and the parents. Hybridization may be a major force driving genomic and transcriptomic changes in newly formed allopolyploids. The formation of allopolyploids may not simply be the sum of hybridization and polyploidization changes but may also involve the interaction of these processes.

## Electronic supplementary material

Figure S1

Figure S2

Figure S3

Table S1

Table S2

Table S3

Table S4

Table S5

Table S6

Table S7

Table S8

Table S9

Table S10

Table S11

Table S12
