# Supplementary figures and images for: Genomic and transcriptomic alterations following intergeneric hybridization and polyploidization in the Chrysanthemum nankingense×Tanacetum vulgare hybrid and allopolyploid (Asteraceae)
Source: Hortic Res. 2018 Feb 7;5:5. doi: 10.1038/s41438-017-0003-0 (PMC5802763; doi:10.1038/s41438-017-0003-0)

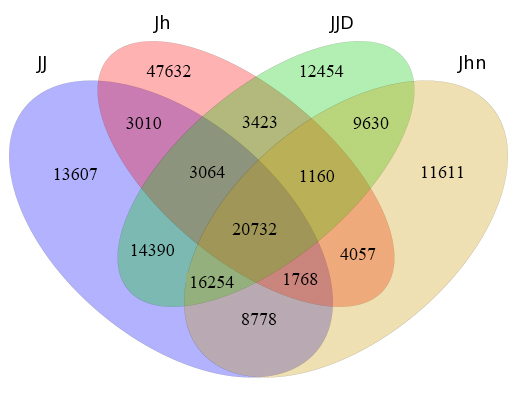

Supplement: Supplementary file 2 — Figure S2 [file 41438_2017_3_MOESM2_ESM.jpg]

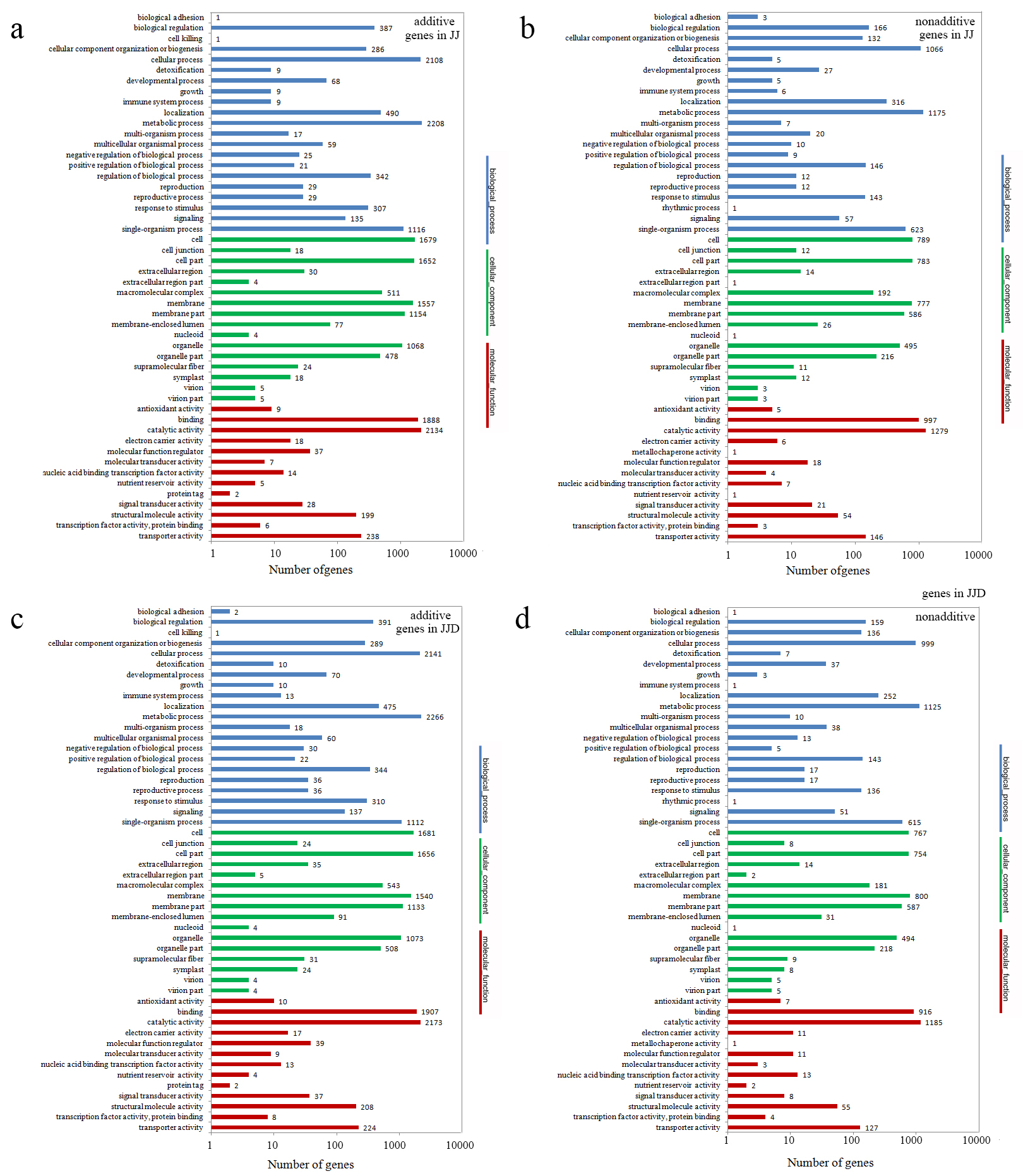

Supplement: Supplementary file 3 — Figure S3 [file 41438_2017_3_MOESM3_ESM.jpg]

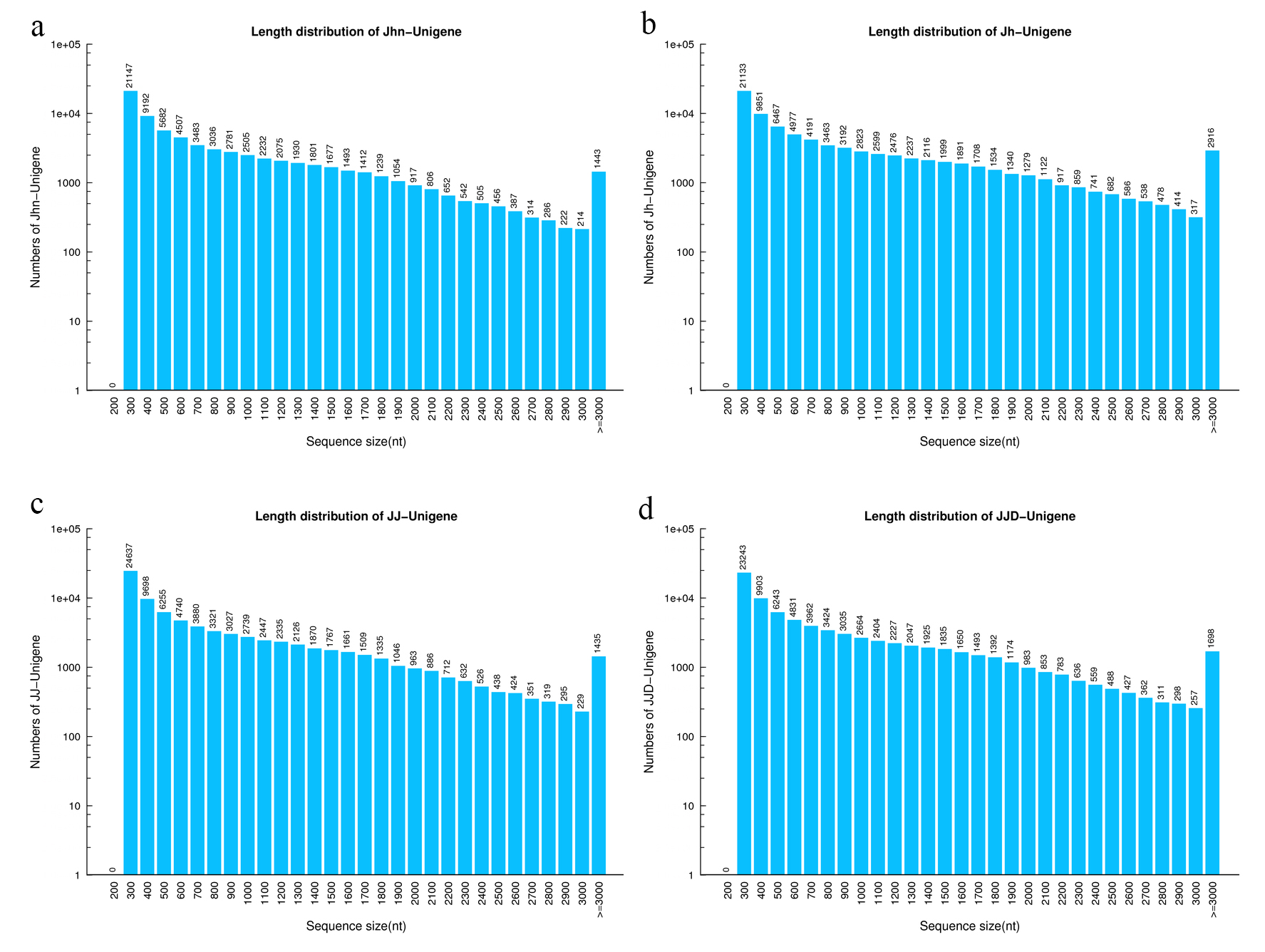

Supplement: Supplementary file 15 — Table S12 [file 41438_2017_3_MOESM15_ESM.xlsx]
