# Supplementary material for: Genomic and transcriptomic alterations following intergeneric hybridization and polyploidization in the Chrysanthemum nankingense×Tanacetum vulgare hybrid and allopolyploid (Asteraceae)
Source: Hortic Res. 2018 Feb 7;5:5. doi: 10.1038/s41438-017-0003-0 (PMC5802763; doi:10.1038/s41438-017-0003-0)
Supplement: Supplementary file 4 — Table S1 [file 41438_2017_3_MOESM4_ESM.docx]

Table S1 Primer sequences used for amplification in SRAP analysis

| Primers | Forward primer | Primers | Reverse primer |
| --- | --- | --- | --- |
| ME1 | TGAGTCCAAACCGGATA | EM1 | GACTGCGTACGAATTAAT |
| ME3 | TGAGTCCAAACCGGAAT | EM2 | GACTGCGTACGAATTTGC |
| ME5 | TGAGTCCAAACCGGAAG | EM4 | GACTGCGTACGAATTTGA |
| ME6 | TGAGTCCAAACCGGTAG | EM5 | GACTGCGTACGAATTAAC |
| ME8 | TGAGTCCAAACCGGTGT | EM6 | GACTGCGTACGAATTGCA |
| ME9 | TGAGTCCAAACCGGTCA | EM7 | GACTGCGTACGAATTATG |
| ME10 | TGAGTCCAAACCGGATG | EM8 | GACTGCGTACGAATTAGC |
| ME11 | TGAGTCCAAACCGGACA | EM9 | GACTGCGTACGAATTACG |
| ME12 | TGAGTCCAAACCGGGAT | EM10 | GACTGCGTACGAATTTAG |
| ME14 | TGAGTCCAAACCGGGCT | EM11 | GACTGCGTACGAATTTCG |
| ME15 | TGAGTCCAAACCGGTGC | EM12 | GACTGCGTACGAATTGTC |
| ME16 | TGAGTCCAAACCGGAGG | EM14 | GACTGCGTACGAATTCAG |
| ME17 | AGCGAGCAAGCCGGTGG | EM15 | GACTGCGTACGAATTCTG |
| ME18 | GAGCGTCGAACCGGATG | EM16 | GACTGCGTACGAATTCGG |
| ME19 | CAAATGTGAACCGGATA | EM17 | GACTGCGTACGAATTCCA |
| ME20 | GAGTATCAACCCGGATT | EM19 | TGTGGTCCGCAAATTTAG |
| ME21 | GTACATAGAACCGGAGT |  |  |
| ME23 | CACAGTCATGCCGGAAT |  |  |
| ME24 | GACCAGTAAACCGGATG |  |  |
