# Supplementary material for: Genomic and transcriptomic alterations following intergeneric hybridization and polyploidization in the Chrysanthemum nankingense×Tanacetum vulgare hybrid and allopolyploid (Asteraceae)
Source: Hortic Res. 2018 Feb 7;5:5. doi: 10.1038/s41438-017-0003-0 (PMC5802763; doi:10.1038/s41438-017-0003-0)
Supplement: Supplementary file 5 — Table S2 [file 41438_2017_3_MOESM5_ESM.docx]

Table S2 Sequences of adaptors and primers used for amplification in MSAP analysis

| Adaptors/primers | Sequence (5'–3') |
| --- | --- |
| *EcoR*I adaptor-1 | CTCGTAGACTGCGTACC |
| *EcoR*I adaptor-2 | AATTGGTACGCAGTCTAC |
| *Hpa*II/*Msp*I adaptor-1 | GATCATGAGTCCTGCT |
| *Hpa*II/*Msp*I adaptor-2 | CGAGCAGGACTCATGA |
| *EcoR*I pre-selective primer | GACTGCGTACCAATTCA |
| *Hpa*II/*Msp*I pre-selective primer | ATCATGAGTCCTGCTCGG |
| *EcoR*I selective primer-1 | GACTGCGTACCAATTCAAC |
| *EcoR*I selective primer-2 | GACTGCGTACCAATTCAAG |
| *EcoR*I selective primer-3 | GACTGCGTACCAATTCAGC |
| *EcoR*I selective primer-4 | GACTGCGTACCAATTCAGG |
| *EcoR*I selective primer-5 | GACTGCGTACCAATTCACA |
| *EcoR*I selective primer-6 | GACTGCGTACCAATTCACG |
| *EcoR*I selective primer-7 | GACTGCGTACCAATTCACC |
| *EcoR*I selective primer-8 | GACTGCGTACCAATTCACT |
| *Hpa*II/*Msp*I selective primer-1 | ATCATGAGTCCTGCTCGGTAA |
| *Hpa*II/*Msp*I selective primer-2 | ATCATGAGTCCTGCTCGGTCC |
| *Hpa*II/*Msp*I selective primer-3  *Hpa*II/*Msp*I selective primer-4  *Hpa*II/*Msp*I selective primer-5 | ATCATGAGTCCTGCTCGGTTC ATCATGAGTCCTGCTCGGTAC ATCATGAGTCCTGCTCGGTGC |
| *Hpa*II/*Msp*I selective primer-6 | ATCATGAGTCCTGCTCGGTAG |
| *Hpa*II/*Msp*I selective primer-7 | ATCATGAGTCCTGCTCGGTTG |
| *Hpa*II/*Msp*I selective primer-8 | ATCATGAGTCCTGCTCGGTCA |
