# Supplementary material for: Genomic and transcriptomic alterations following intergeneric hybridization and polyploidization in the Chrysanthemum nankingense×Tanacetum vulgare hybrid and allopolyploid (Asteraceae)
Source: Hortic Res. 2018 Feb 7;5:5. doi: 10.1038/s41438-017-0003-0 (PMC5802763; doi:10.1038/s41438-017-0003-0)
Supplement: Supplementary file 6 — Table S3 [file 41438_2017_3_MOESM6_ESM.docx]

| Gene ID | Forward primer | Reverse primer |
| --- | --- | --- |
| Unigene50220_JJ | CAAGGTGATGATGAGGAGCA | ACTGTGTAGCAGCCATTGGA |
| Unigene4054_JJ | GGAGGAAGTGGGGACGGT | CTGAGGCGTGTTACTGGACTG |
| Unigene3261_JJ | GGTTTTCAATCCTCTTCGTCATC | CAAGCCAAGCTCCAATCCAG |
| CL10525.Contig2_JJ | GAAAGTCGTCCTCTTACCACCTC | CGCCCCTTCTCAACCCAT |
| CL8971.Contig2_JJ | TGTCTGCGAGATTTCTGGATACG | TTCGGAAGGGGAAAGCAAT |
| Unigene16009_JJ | GGTGGGGCAAGCTAGAGCG | CTGAGCATGTATGGTATGGAAAACAGTATC |
| CL10301.Contig4_JJ | GTGCTGAAATCGGTGAGGG | CTTGTGCTAACTGCATCATGTCA |
| Unigene30090_JJ | ACACTAATACCTCGTCATTCTCCAA | CTTCCATTTATCCAACGGCTC |
| Unigene34986_JJ | CGATGTCGTCACTGGTAAAGG | ATTGTCCTAAACCTCCCATAACC |
| Unigene7880_JJ | TGCTTTTGACGAGTATTCAGCTC | TTTTGAGGTCTCCGTCTTTAGGT |
| Unigene30016_JJ | GGTGGTAAGAAGAAAACTCAACGC | GCTCCGACAACCCCGATAC |
| Unigene16464_JJ | GATGGCGGCTGCGGCTTAT | GGCGTGGGACACTCAGGAAT |
| EF1α | TTTTGGTATCTGGTCCTGGAG | CAAGTTGCTCAATATCTCCACAGA |

Table S3 Primer sequences for qRT-PCR
